# Should the findings of the DAPT trial change my practice?

**Published:** 2016

One of AfricaPCR 2016’s final sessions discussed this question and a distinguished panel concluded, albeit with a few reservations and caveats, that the answer is ‘yes’. DAPT was the first large trial to evaluate the benefits of dual antiplatelet therapy prolonged beyond 12 months after percutaneous coronary intervention (PCI).

DAPT set out to investigate, as an effectiveness endpoint, whether prolonged dual antiplatelet therapy was associated with a reduction in stent thrombosis and in major cardiovascular and cerebrovascular events. The safety endpoint was its effect on moderate or severe bleeding.

It found that prolonging therapy up to 30 months was associated with a 71% relative risk reduction in stent thrombosis and a 29% relative risk reduction in major events. But there was a price to pay in that there was a 1.6–2.5% increase in rate of bleeding.

Prior to DAPT, previous practice had been to give therapy for much shorter periods, usually one to six months. Reviewing what was known before the study, Dr Colin Schamroth, from Johannesburg, noted that most previous studies had suggested that there was no solid evidence to support treatment beyond 12 months. TRITON-TIMI had hinted that there might be a case for prolonging therapy, but had still concluded that most benefit was accrued over 12 months.

The 2012 European guidelines currently recommend dual antiplatelet therapy for a minimum of one month in patients with bare-metal stents and six months in those with drugeluting stents, with three to six months for the former and six to 12 months for the latter being the preferred strategy. Drugeluting stents are associated with higher rates of thrombosis. Twelve months of therapy are recommended for unstented STEMI patients.

Drilling down into the details of DAPT, Dr Riaz Dawood, also from Johannesburg, noted that it was a well-designed trial with results that were valid and not underpowered. It included nearly 10 000 patients from 11 countries. However, none of these was in Africa, South America or Asia. He also underscored that it was a low-risk population, excluding patients with a high risk of bleeding or ischaemic events.

The 29% relative risk reduction in major events was driven mainly by a decrease in myocardial infarction (MI), with no significant differences observed in respect of mortality and haemorrhagic stroke. The increase in bleeding risk was driven by moderate bleeding, with severe, life-threatening bleeding not significant. All-cause mortality, however, showed a 36% increase, but this was not driven by either cardiovascular death or by bleeding, but rather by cancer. Dr Dawood felt that this was most likely a ‘play of chance’, unrelated to dual antiplatelet therapy.

In conclusion, DAPT found that prolonging therapy up to 30 months was associated with a significant reduction in rates of thrombosis and major events, notably MI, but at a risk of increased rates of bleeding. The MI benefit was not limited to stented sites. The findings were also consistent, regardless of which thienopyridine agents were used.

So should these findings change clinical practice? Prof Pravin Manga, from Johannesburg, offered a cautious ‘yes’. ‘The evidence suggests that longer and maybe even indefinite therapy may be of benefit, but we need to be very careful when it comes to patient selection.’

Dr Dawood echoed this. ‘Bleeding worries us more than ischaemic events, and bleeding risk increases with age, so one needs to be especially cautious in elderly patients. But in patients without contra-indications who are at risk of thrombosis, but with a low bleeding risk, why not continue?’

